# Demographic and clinical features of dengue fever infection in Pakistan: a cross-sectional epidemiological study

**DOI:** 10.1186/s40794-024-00221-4

**Published:** 2024-04-05

**Authors:** Tanzeel Zohra, Misbahud Din, Aamer Ikram, Adnan Bashir, Haroon Jahangir, Imran Sikandar Baloch, Sundas Irshad, Abdul Waris, Muhammad Salman, Somia Iqtadar, Muhammad Ayaz

**Affiliations:** 1 National Institutes of Health, Islamabad, 45500 Pakistan; 2 Quaid-I-Azam University, Islamabad, 45320 Pakistan; 3 Health Information Systems Program, Islamabad, Pakistan; 4 Primary and Secondary Healthcare Department, Government of Punjab, Lahore, Pakistan; 5 Dengue Expert Advisory Group Punjab, King Edward Medical University, Lahore, 54000 Pakistan; 6 Department of Pharmacy, University of Malakand Khyber, Chakdara, 18800 Pakistan

**Keywords:** Dengue, Epidemiology, Antibodies, NS1, Punjab

## Abstract

**Background:**

Dengue fever caused by dengue virus is a tropical disease and is among the deadliest vector-borne diseases. The humid and hot summers of Pakistan support the probation of the vectors responsible for the transmission of viral and other parasitic diseases.

**Methodology:**

A retrospective study, from 2012- 2019, of dengue infected individuals from the Punjab province of Pakistan was carried out to analyze epidemiology, clinical and laboratory findings of subjects with dengue virus infection. Data was derived from National Institute of Health (NIH) followed by Dengue control program of Pakistan, covering the incidence rate in 36 districts of Punjab and Islamabad Capital Territory (ICT) respectively. Patients data including the presence of dengue specific antigen or/and antibodies such as NS1 and IgG/IgM were observed. The study also included the analysis of demographic data, geographic data, and the month-wise distribution of dengue cases to examine seasonal trends.

**Results:**

We analyzed 25,682 dengue infected individuals. The statistical analysis revealed a significant association between genders in which male population was more affected by dengue than females. It was also noted that the middle age group was the most affected age group while the highest number of cases were reported in October. Rawalpindi and Lahore were the most affected cities in Punjab province while Islamabad represented the highest number of cases during the recent outbreak in 2019. The IgM and IgG antibodies were highly prevalent among the infected patients.

**Conclusion:**

Dengue is endemic in Pakistan, circulating throughout the year. Highest number of cases were observed in the month of October, September and November respectively. Association between climate change and vector-borne diseases need to be investigated in Pakistan as they significantly influence the timing and intensity of dengue and other disease outbreaks. Further exploration of hematological parameters is required to better diagnose and treat the disease. For the effective control of dengue outbreaks, awareness campaigns on sewage management and vector control along with social factors are strongly recommended for better control and eradication of the disease.

## Introduction

Dengue is a deadly emerging and re-emerging viral infection, which is prevalent in the tropical and subtropical regions throughout the globe [1, 2]. The mortality and morbidity of Dengue fever has made it a major public health concern. Dengue fever is caused by the dengue virus (DENV), a single-stranded RNA virus that belongs to the genus *Flavivirus* of the family Flaviviridae in Amarillovirales order. DENV has been classified into four distinct serotypes (DEN-1, DEN-2, DEN-3, DEN-4) and ten different genotypes based on the variation of sequence in the virus's envelope gene [3, 4]. Two species of A*edes* mosquito, *Aedes aegypti* (*A. aegypti*) and *Aedes albopictus* (*A. albopictus* are mainly responsible for the transmission of the dengue virus) [5, 6]. The dengue virus infection is categorized into three different classes ranging from asymptomatic and moderate dengue fever to severe dengue shock syndrome (DSS) and dengue hemorrhagic fever (DHF), which may lead to death [7, 8]. The normal mortality rate of DENV infection is less than 1%; however, it usually stands 1–5% when there is no proper management, care and clinical administration [9, 10]. According to the World Health Organization (WHO), half of the world population is now at risk because of the dramatic growth of DENV infection incidence in recent decades. DENV infects approximately 100–400 million individuals each year throughout the world [11].

In Pakistan, the DENV is endemic since 1995 due to temperate climate of the country [12, 13]. Pakistan is located in South Asia between longitudes 61° and 75.5° east, latitudes 23.45° and 36.75° north, and the sixth most populated country of the world [14]. Several vector-borne diseases, including leishmaniases, dengue hemorrhagic fever, West Nile virus infection, malaria, scrub typhus, typhus, Crimean-Congo hemorrhagic fever, and Japanese encephalitis, have been reported in the region. These occurrences can be attributed to the subtropical location and favorable climatic conditions for vectors. [1, 14, 15]. In 1994, the first outbreak of dengue fever due to the serotypes DEN-1 and DEN-2 was reported, which led to thousands of morbidities [13]. A second outbreak involving DEN-3 serotype occurred in Karachi in 2005 with the dramatic increase of severe DHF patients [16]. Since 2006, every year, the dengue outbreaks and co-circulation of various dengue serotypes have been reported. In Lahore, Punjab, a major epidemic occurred in 2011 which reported more than 50,000 cases, this was followed by a huge dengue outbreak in Khyber Pakhtunkhwa province of Pakistan in 2013 [2, 17, 18]. Due to the rich fauna, artificial water reservoirs for various purposes, floods from heavy rainfall, open irrigation channels, and vast agricultural land of Pakistan provide a plenty of breeding sites for mosquito vector (s) [2]. The activities of dengue virus vectors vary according to seasons, various geographical areas of the country. It has been observed that incidence of cases typically increases after the rainy seasons [2, 8]. Primary exposure to the DENV infection leads to the development of lifelong immunity against the particular serotype, while secondary infection may lead to the development of life-threatening conditions of the disease such as DSS and DHF [4, 19]. A specific type of antibody, known as immune globulin, can be produced in response to infection by the same serotype of DENV.. However, the DSS and DHF mediated by an antibody-dependent enhancement (ADE) mechanism might occur if the causative agent is a different serotype [20].

The current study was aimed to examine the epidemiological and serological characteristics of dengue virus infection in Punjab province of Pakistan. Punjab is the most populous province of the Pakistan with diverse range of climate conditions. We examined the dengue positive patients from 2012 to 2019 from Punjab, outside Punjab and Islamabad.

## Materials and methods

### Selection of study area

**Fig. 1:**
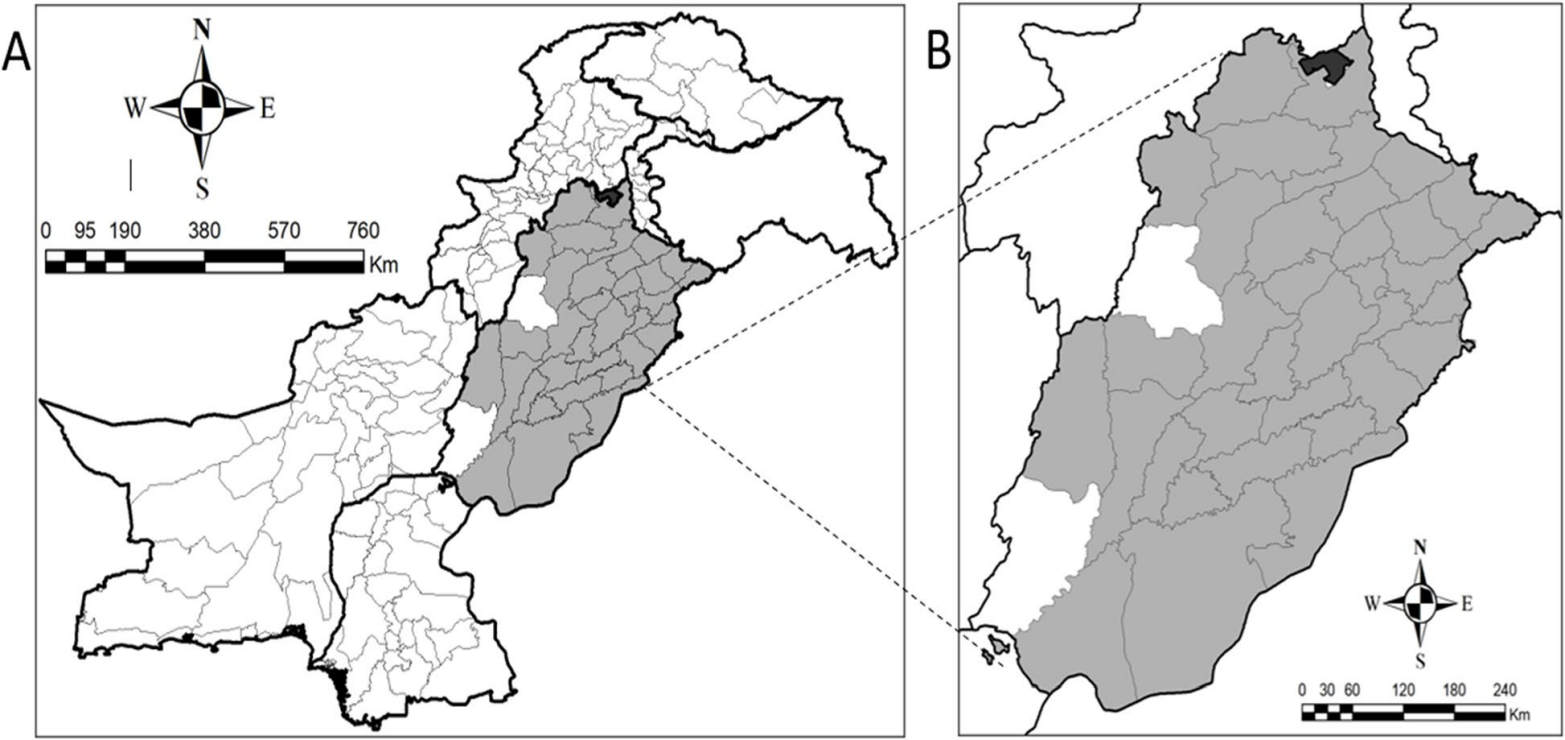
Map of Pakistan with Punjab province in dark gray (**A**) and map shows Islamabad capital territory (ICT) in black color and Punjab province with its different districts in dark grey where dengue infected patients reported (**B**). The black bold lines show the boundaries of regions/Provinces of Pakistan

Based on population, Punjab is the largest Province of Pakistan that is situated in northwestern region of Pakistan and divided in 36 districts. So this cross-sectional retrospective study was conducted in Punjab, and federally administered, a capital territory of Pakistan i.e. Islamabad from 2012–2019 and includes 34 districts of Punjab i.e. Attock, Bahawalnagar, Bahawalpur, Chakwal, Chiniot, Dera Ghazi Khan, Faisalabad, Gujranwala, Gujrat, Hafizabad, Kasur, Khanewal, Khushab, Lahore, Layyah, Lodhran, Mandi Bahuddin, Multan, Muzaffargarh, Nankana Sahib, Narowal, Okara, Pakpattan, Rahim Yar Khan, Rawalpindi, Sahiwal, Sargodha, Sheikhupura, Sialkot, Toba Tek Singh, Vehari, Jhang, Jehlum and Mianwali to examine epidemiological and clinical features of Dengue virus infection (Fig. 1).

### Data source

Since 2010, Pakistan has been experiencing an epidemic of dengue fever. In this study, the dengue epidemiological data from 2012 to 2019 was derived from National Institute of Health followed by Dengue control program of Pakistan, covering the incidence rate in 36 districts of Punjab and ICT respectively. The suspected patients were examined for the presence of IgG, IgM or/and NS1 antigen. The patients examined positive for the presence of either antigen (Ag), antibodies (Abs) and/or both were included in this study. The demographic information of individuals including age, gender, occupation etc. were recorded. The data regarding sign and symptoms of dengue infection (abdominal pain, enlarged liver, fever, haematemesis, skin rashes, nose and gum bleeding), previous exposure, hospital stay etc. was also recorded.

### Inclusive and exclusive criteria

This study includes positive dengue patients data particularly and covering all districts of Punjab and Islamabad capital territory (ICT) respectively. In the study, 26, 582 positive patients data from 34 districts of Punjab, outside of Punjab (not resident of Punjab, who only come from other regions of Pakistan to visit during selected time period) and Islamabad territory has been selected as a sample size (n) for further epidemiological and serological analysis.

### Ethical approval

The study was approved by National Institute of Health Islamabad. There was neither any sort of study-related patient interaction or interventions was involved. Furthermore, all data was analyzed anonymously and henceforth, informed consent was not prerequisite.

### Case definition and laboratory investigation

Positive cases are diagnosed based on epidemiological exposure and clinical manifestation by experienced doctors and further confirmed by relevant laboratory results, such as enzyme linked immunosorbent assay (ELISA), NS1 antigen or polymerase chain reaction.

Based on dengue antibodies or NS1 antigen presence, the dengue infection has been classified as primary or secondary infection. Primary infection is defined as an IgM-negative/IgG-negative; or IgM-positive/IgG-negative on the blood sample drawn within 3 days of symptom onset. Secondary infection was defined as an IgM-negative/IgG-positive or an IgM-positive/IgG-positive result on the blood sample drawn within 3 days of symptom onset.The titers, of IgM and IgG antibodies vary based on whether the infection is primary or secondary. In the case of a primary (first) dengue infection, IgM levels are notably elevated, whereas in a secondary infection, IgM levels tend to be lower. Conversely, the levels of IgG increase during a secondary infection A patient was considered positive for dengue infection upon the detection of anti-dengue IgM. Similarly, NS1 is also considered an important biomarker for detection of dengue infection and hence was used for the rapid detection. The infection was also considered primary infection when the patients was negative for IgG and IgM antibodies but positive for NS1 antigen.

### Data analysis

Different statistical tools were used for the analysis of data. The t-test and Mann–Whitney U test were used. Different categorical variables were expressed as percentage and frequency rate. Fisher’s exact test and χ^2^ test were used for the analysis of categorical variables. Various comparisons among different groups were also performed using the Bonferroni adjustment method. *P*-value of ≤ 0.05 was considered statistically significant. The maps were created using Arc View Geographical Information Software3 (Arc GIS). Statistical Package for the Social Sciences (SPPS) (version 23.0) was used for all the statistical analyses.

## Results

The current study included 26,582 individuals who were tested positive for anti-dengue antibodies or NS1 antigen from 2012 to 2019 in Punjab province of Pakistan. Among the total infected individuals, 7,301 (27.46%) were female while 18,381 (69.14%) were male. However, the 900 (3.38%) individuals had an unknown sex.The dengue positive gender-wise cases along with years is represented in Table 1. Statistical analysis revealed a significant association (*p* value < 0.001) dengue infection and gender. The ratio of male-infected individuals was higher (18,281, *n* = 25,682) than female individuals (7,301, *n* = 25,682).

**Table 1:** The gender-wise dengue infected individuals in different years are represented

Year	Females	Males	*P* value
2012	12	28	< 0.001*
2013	881	1766	
2014	470	973	
2015	1246	2966	
2016	1463	3590	
2017	309	726	
2018	219	742	
2019	2701	7590	
**Total**	7301	18,381	

The chi-square test was applied and *p* < 0.05 was considered significant at a 95% confidence interval

^*^p-value is significant

### Dengue infection in different age groups

The age-wise cases are represented in Table 2. We observed that the middle age group (21 to 30 years) was the most affected group (8,261, *n* = 25,682) followed by 11 to 20 years age group (5,551, *n* = 25,682) and 31 to 40 years age group (5,339, 25,682) respectively. The highest number of cases (*n* = 10,288) were reported in the year 2019. The older individuals (> 70 years) were found to be the least affected age group (*n* = 238) followed by smallest age group (1 to 10 years) with 701 patients and age group 60 to 70 years with 721 cases. The statistical analysis revealed a significant association (*p* value < 0.001) between age group and dengue infection.

**Table 2:** Age-wise cases of dengue virus infected individuals are represented

Age group	2012	2013	2014	2015	2016	2017	2018	2019	Total	*p* value
1 to 10	3	140	43	124	113	23	30	225	701	< 0.001*
11 to 20	9	631	401	950	991	205	195	2169	5551	
21 to 30	19	818	448	1323	1653	337	300	3363	8261	
31 to 40	5	519	269	848	1024	231	227	2216	5339	
41 to 50	2	312	167	525	653	118	126	1273	3176	
51 to 60	1	129	73	295	385	76	59	677	1695	
60 to 70	1	72	24	116	176	33	20	279	721	
> 70		27	15	34	59	12	5	86	238	
**Total**	**40**	**2648**	**1440**	**4215**	**5054**	**1035**	**962**	**10,288**	**25,682**	

The chi-square test was applied and *p* < 0.05 was considered significant at a 95% confidence interval

^*^p-value is significant

### Clinical features and serological pattern of dengue infection

The clinical features revealed that fever was the most common symptom observed in individuals of all age groups and both genders. The other common symptoms included headache, fatigue, nose and gum bleeding, abdominal pain, hematemesis, skin rashes and vomiting. The patients were categorized positive for dengue virus infection based on serological analysis. The presence of anti-dengue antibodies such as IgG and/or IgM along with NS1 antigen were examined. We found that IgM antibody was the most prevalent anti-dengue antibody detected in 11,148 individuals including 7,679 males while female 3,469 individuals. Anti-dengue specific IgG antibodies were found to be the second most (*n* = 5,396, males = 4,155, females = 1,241) prevalent serological marker after anti-dengue IgM. The serological pattern observed in our study is represented in Table 3. A total of 1,313 individuals were positive for both IgG and IgM including 955 males while 358 females. We observed that 2,649 individuals were positive for both types of anti-dengue antibodies while also for NS1 antigen as represented in Table 3.

**Table 3:** Serological outcomes of patients are represented. The IgG presence shows the secondary dengue infection and IgM presence shows primary dengue infection. Whereas both presences show primary and secondary infection at a time

Anti-dengue antibodies/ antigen	Male	Female	Total
IgG	4,155	1,241	5,396
IgM	7,679	3,469	11,148
NS1	1,939	587	2,526
IgG + IgM	955	358	1,313
IgG + NS1	1,141	191	1,332
NS1 + IgM	1001	317	1,318
NS1 + IgG + IgM	2261	388	2649
Total			25,682

### Region-wide distribution of dengue infection

Dengue positive individuals in various region/district of the Punjab province along with cases of outside of Punjab province are reported in Table 4. We have also summarized the cases of Capital territory Islamabad in Table 4. The data reveals that Rawalpindi (*n* = 7,215 cases) was the more affected region followed by Lahore (*n* = 2,958 cases) and Multan (*n* = 386 cases). It is important to mention that the capital territory of Islamabad alone represented the highest number of cases. We observed more than 10, 000 dengue positive cases in 2019 alone in Islamabad. We also reported 709 cases of those patients that were diagnosed in Punjab; however, they were resident of the Punjab province, represented in Table 4. Some of the regions represented the lowest number of cases such as Layyah, Khushab, and Jhelum which might be due their favorable climate condition.

**Table 4:** District wise dengue positive cases in Punjab province

District	2012	2013	2014	2015	2016	2017	2018	2019	Total
Attock	-	3	12	19	23	15	13	-	85
Bahawalnagar	1	4	-	1	6	2	2	-	16
Bahawalpur	-	16	2	6	12	1	2	-	39
Chakwal	-	4	4	7	11	12	42	-	80
Chiniot	-	2	1	2	-	2	-	-	7
Dera Ghazi Khan	-	3	-	1	4	4	1	-	13
Faisalabad	12	21	1	12	100	9	12	-	167
Gujranwala	1	6	4	3	11	14	9	-	48
Gujrat	-	2	3	1	2	2	-	-	10
Hafizabad	1	2	-	-	1	2	2	-	8
Kasur	-	13	-	-	15	3	-	-	31
Khanewal	-	3	-	2	5	2	1	-	13
Khushab	-	1	-	-	1	2	-	-	4
Lahore	10	1510	97	142	1108	74	17	-	2958
Layyah	1	1	-	-	-	-	1	-	3
Lodhran	-	2	-	1	3	-	2	-	8
Mandi Bahuddin	-	4	4	2	3	-	-	-	13
Multan	-	9	2	362	6	3	4	-	386
Muzaffargarh	-	2	-	9	6	3	-	-	20
Nankana Sahib	1	1	1	1	7	4	-	-	15
Narowal	1	6		4	8	2	2	-	23
Okara	1	3	2	-	2	1	-	-	9
Outside Punjab	-	21	22	322	150	132	62	-	709
Pakpattan	1	4	1	1	4	1	-	-	
Rahim Yar Khan	-	3	1	1	10	2	-	-	17
Rawalpindi	-	893	1212	3194	1159	334	423	-	7215
Sahiwal	-	6	1	4	7	-	1	-	19
Sargodha	1	5	3	2	18	5	2	-	36
Sheikhupura	4	80	60	10	25	8	1	-	188
Sialkot	-	5	1	-	4	1	1	-	12
Toba Tek Singh	-	4	1	4	8	1	4	-	22
Vehari	-	5		4	6	-	2	-	17
Jhang	4	-	-	1	11	1	-	-	17
Jehlum	2	-	-	2	-	-	-	-	4
Mianwali	1	-	-	1	2	2	5	-	11
Islamabad	-	-	95	-	2314	393	353	10,292	13,447

### Month-wise distribution of dengue cases

**Fig. 2:**
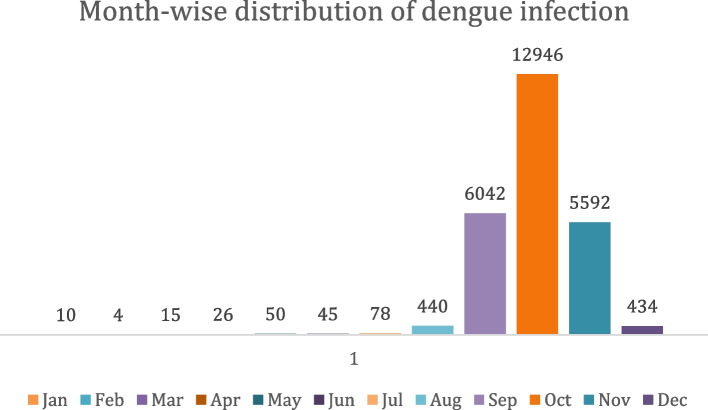
Month-wise dengue cases are represented. October represented the highest number of cases

The month-wise pattern of dengue cases is represented in Fig. 2. Highest number of cases were observed in the month October (*n* = 12,946) followed by September (*n* = 6,042), and November (*n* = 5,592). Similar cases were recorded in August and December which were 440 and 434, respectively. The lowest number of cases were recorded in winter which steadily increased from February (*n* = 4) to March (*n* = 15), April (*n* = 26), May (*n* = 50) and then July (*n* = 78). A slight decrease in cases was observed in June (*n* = 45) which could be linked to the increased temperature experienced in June.

## Discussion

Dengue, a vector-borne viral disease that infects about 100 to 400 million individuals annually around the globe [21]. The burden of dengue virus infection has increased alarmingly around the globe in recent years. Although the risk of dengue infection exists in more than 128 countries, about 70% cases are reported from Asia [22, 23]. The number of cases reported to the World Health Organization in the last two decades has increased by more than eight-fold. The humid and hot summers of Pakistan increase the propagation of *Aedes* species that have been known vectors for dengue virus transmission. The dengue virus infection was identified in 1994 in Pakistan, although cases were experienced in 1982, however no information was available at that time [24]. Thousands of cases were reported in Karachi in 2005 and later in the entire country in 2010 [25].

In our study, we examined the epidemiological and serological features of dengue virus infection in the Punjab, the most populous province of Pakistan. We observed 25,682 dengue infected individuals in which 7,301 were female while 18,381 were male individuals and the statistical analysis revealed significant association between the gender and dengue infection. Our results are in accordance with the previous studies which reported higher risk of dengue infection in male individuals in other regions of the country [4, 26, 27]. Higher prevalence in male individuals could be possibly linked to the lifestyle of male individuals and their increased exposure to the environment in our society. The age-wise distribution of the cases is represented in Table 2. The middle age group (21 to 30 years) was observed as the most affected group. The high frequency of cases in the age group of 21 to 30 years in this study was due to larger population size in that age category. The statistical analysis revealed a significant association (*p* value < 0.001) between age group and dengue infection. Previous studies have also reported the young population as the most vulnerable group to viral infections which could also be linked to their exposure to the environment. The increased incidence in the populous districts such as Rawalpindi and Lahore as represented in Table 4 could be linked to the standing of these regions in trade and industrial activities along with climate conditions and more testing facilities. The highest number of cases were reported from Rawalpindi which is situated within the federal capital territory of Islamabad and is considered as hub for trades and business. Another reason could be travel of individuals from rural areas to urban areas. Some of the region represented the lowest number of cases such as Jhelum, Khushab, and Rahim Yar Khan which are known for extreme temperatures. The extreme temperature could also be linked with the lower number of cases in such regions which could affect the propagation of vectors responsible for dengue transmission. Previous studies have reported that climate variation can potentially increase the dengue transmission via the alteration in vector’s prevalence as well as changes in human behavior [28, 29]. Dengue outbreaks have been observed to be associated with climate change globally [30]. The main determinant of climate variability in Asia are the monsoons. It has been reported that dengue outbreaks have significantly coincided with the post-monsoon season of variable rainfall and vectors of dengue virus are more abundant in the post-monsoon season [31].

Among the clinical features, fever was the most common symptom along with headache, fatigue, nose and gum bleeding, abdominal pain, hematemesis, skin rashes and vomiting. The patients were categorized positive for dengue virus infection based on serological analysis. The presence of anti-dengue antibodies such as IgG and/or IgM along with NS1 antigen are represented in Table 3.

The highest number of cases were observed in the year 2019 in Islamabad as represented in Table 4. Previously, the highest number of dengue cases have been reported in the same year worldwide [32]. Our study suggests the immediate proper monitoring of epidemiological, serological and clinical features of dengue infection in the regions. The co-epidemics of dengue infection with other viral infections such as Covid-19 and polio could aggravate the less developed and overburdened health system of Pakistan countries [33–35]. For the effective control of dengue outbreaks, social factors along with sewage management and vector control are strongly recommended. We also suggest an awareness campaign on the management, control, and eradication of such diseases.

## Conclusion

From the current study, it could be concluded that dengue infection was increased in Pakistan. Male individuals and the young population were the most vulnerable group to dengue infection. The federal capital represented the highest number of cases in 2019. Rawalpindi and Lahore were the most affected regions of the Punjab province while highest number of cases were reported in October. Further investigations are needed to explore the elevation in hematological and other parameters associated with dengue virus infection. The study limitations include the usage of antibodies based detections i.e. IgM detection for diagnosis of dengue which might have cross-reactivity related issues between other circulating flaviviruses. Further exploration of hematological parameters is required to better diagnose and treat the disease. We also suggest that the link between climate change and vector-borne diseases need to be investigated in Pakistan as they significantly influence the timing and intensity of dengue and other disease outbreaks. Public awareness and preventive strategies are highly recommended to overcome the burden of viral diseases including dengue, polio and Covid-19.

## Data Availability

Data related to this manuscript is available to researchers from Tanzeel Zohra upon request.
